# Temporal patterns of energy equivalence in temperate soil invertebrates

**DOI:** 10.1007/s00442-015-3317-3

**Published:** 2015-04-23

**Authors:** Werner Ulrich, Alexia Hoste-Danyłow, Katarzyna Faleńczyk-Koziróg, Izabela Hajdamowicz, Krassimira Ilieva-Makulec, Izabella Olejniczak, Marzena Stańska, Jolanta Wytwer

**Affiliations:** Nicolaus Copernicus University, Toruń, Poland; Institute of Ecology and Bioethics, Cardinal Stefan Wyszyński University, Warsaw, Poland; Kazimierz Wielki University, Bydgoszcz, Poland; Siedlce University of Natural Sciences and Humanities, Siedlce, Poland; Museum and Institute of Zoology, Warsaw, Poland

**Keywords:** Edaphon, Equal biomass hypothesis, Metabolic theory, Trophic group, Allometric scaling

## Abstract

The question whether total population energy use is invariant to species body size (the energy equivalence hypothesis) is central to metabolic ecology and continues to be controversial. While recent comparative field work and meta-analyses pointed to systematic deviations of the underlying allometric scaling laws from predictions of metabolic theory none of these studies included the variability of metabolic scaling in ecological time. Here we used extensive data on the invertebrate soil fauna of Kampinos National Park (Poland) obtained from six consecutive quantitative sampling seasons to show that phylogenetically corrected species density—body weight and population energy use—body weight relationships across all soil fauna species and within trophic groups and body weight classes were highly variable in time. On average, population energy use tended to increase with species body weight in decomposers and phytophages, but not in predators. Despite these trends, our data do not exclude the possibility that energy equivalence marks the central tendency of energy use in the edaphon. Our results highlight the need for long-term studies on energy use to unequivocally assess predictions of metabolic theory.

**Electronic supplementary material:**

The online version of this article (doi:10.1007/s00442-015-3317-3) contains supplementary material, which is available to authorized users.

## Introduction

The energy equivalence rule (Damuth 1981) states that the total energy use of different populations relying on the same source of energy is independent of the average body weight of the members of each population. Damuth (1987) derived this rule from the notion that the allometric scaling of individual energy use (*M*) and population density (*D*) on average body weight (*W*) [the metabolic rate—body weight (MWR) and density—body weight relationships DWR)] have opposite directions and similar scaling exponents, *u* ≈ *v*:

1 $$M\,\alpha \,W^{u}$$

2 $$D \,\alpha \,W^{ - v} .$$

Consequently, the total population energy use, calculated as the product of individual energy use and population density (=*M* × *D*), should be invariant of body mass

3 $$MD\,\alpha \,W^{u} W^{ - v} = W^{z \approx 0} .$$

In contrast to warm-blooded species, where a large part of energy consumption goes into heat production, most cold-blooded invertebrates use the major part of the consumed energy for individual growth. Consequently, the equal biomass hypothesis (Sheldon et al. 1972; Brown and Maurer 1988) states that total population biomass (*B*) of poikilothermous species should also be roughly invariant of individual body weight.

4 $$B = WD = W^{y \approx 0}$$

Since its introduction, the energy equivalence rule (EER) has been criticized on empirical (e.g. Griffiths 1992; Blackburn et al. 1993; Russo et al. 2003; Ehnes et al. 2014), theoretical (Marquet et al. 1995; Glazier 2005; Martinez del Rio 2008), and methodological (Medel et al. 1995; Isaac and Carbone 2010) grounds and still remains highly controversial (e.g. Damuth 2007; Hayward et al. 2009; DeLong 2011; Isaac et al. 2011; Munn et al. 2013; Ehnes et al. 2014; Sechi et al. 2015). Based on the empirical observation that metabolic rate scales to body weight with an exponent of *u* = ¾ (e.g. Kleiber 1932; Peters 1983; Savage et al. 2004; Farrell-Gray and Gotelli 2005) earlier tests of the EER concentrated on the relationship between population density and body mass and assessed whether the scaling exponent approximately equalled −¾. However, there is no consensus that MWR and DWR three-quarter exponents are universal or at least mark the central tendency of the allometric scaling of population density with body weight (reviewed in Glazier 2005; Reuman et al. 2009; Isaac and Carbone 2010; Sechi et al. 2015).

Several authors pointed to lower DWR scaling exponents at higher trophic levels due to decreasing resource availability (Brown and Gillooly 2003; Ehnes et al. 2014). Additionally, Chown et al. (2007) and Ehnes et al. (2011) found taxon-specific scaling exponents in arthropods and soil invertebrates. Both findings contrast to the generality of the EER. However, taxon-specific variability in DWR and MWR scaling are expected if these reflect adaptations to the specific habitat requirements and life history strategies of focal taxa (Hechinger et al. 2011) or to habitat-specific resource limitations (Ott et al. 2014). This is best seen in arthropods where larval energy use alone might strongly deviate from EER predictions, while EER tests generally focus on adults due to taxonomical difficulties in larval determination. Therefore, taxon-specific deviations from the EER prediction do not exclude the applicability of EER across taxon and trophic levels if energy equivalence is the central tendency of total energy use within a habitat (Farrell-Gray and Gotelli 2005). Indeed, recent tests of EER that focused on comparisons of MWR and DWR scaling slopes within the framework of metabolic theory (Brown et al. 2004; Deng et al. 2008) returned either a broad accordance of the energy consumption of global soil animals with EER (Savage et al. 2004; Mulder et al. 2005; Meehan 2006a, b; Hechinger et al. 2011) or a rejection of EER for various soil taxa in temperate forests (Ehnes et al. 2014; Ott et al. 2014) and grasslands (Sechi et al. 2015).

In this respect, it is important to note that using smaller taxa or trophic groups instead of the whole fauna within a given habitat generally reduces the absolute difference in abundance between the species. It has long been noted (McNab 1988; Hayward et al. 2009) that a low variability in the x-variable generally decreases the slopes of allometric regressions. Therefore, tests for EER regression slopes should control for the variability in body weight included in the regression (Savage et al. 2004; Farrell-Gray and Gotelli 2005). Apparently, differences in body weight variability might explain at least a part of the contrasting results reported by the global surveys of, for example, Brown et al. (2004), Farrell-Gray and Gotelli (2005), and Meehan (2006a) and the local data of, for example, Blackburn et al. (1993), Chown et al. (2007), and Ehnes et al. (2014).

A second point that has been neglected in most studies on allometric body weight scaling regards phylogenetic non-independence. Although White and Seymour (2003) and Bokma (2004) addressed this problem with respect to slope estimates, few studies used appropriate methods to correct for phylogenetic relatedness in allometric studies (reviewed in Capellini et al. 2010). Earlier studies either ignored phylogenetic effects (Brown et al. 2004; Savage et al. 2004) or tried to reduce phylogenetic non-independence by separate analyses of broader taxa or trophic groups (e.g. White and Seymour 2003; Mulder et al. 2011; Ehnes et al. 2014). George-Nascimento et al. (2004), Duncan et al. (2007), and Raichlen et al. (2011) found a better agreement of data with the EER predictions after accounting for the phylogenetic non-independence of the taxa involved. In turn, Capellini et al. (2010) reported taxon-specific mammal metabolic scaling exponents that deviated from theory. To our knowledge tests of EER using phylogenetic explicit methods based on phylogenetic trees have not been conducted. Therefore, we hypothesize that part of the contrasting results regarding energy equivalence might stem from inadequate correction for phylogenetic non-independence.

Third, previous studies on EER regression used (averaged) temporal point data, either obtained from literature sources (e.g. Savage et al. 2004; Capellini et al. 2010) or short-term field observations (e.g. Meehan et al. 2006, Mulder et al. 2011). However, as estimates of population energy use (Eq. 3) and biomass (Eq. 4) are calculated from observed local abundances that generally vary considerably in time we expect also a high variability in the estimates of population energy use. In this case most tests of EER might return deviations from energy equivalence, indicating taxon- and habitat-specific patterns, even if EER were the central tendency in time.

Below we use an extensive and taxonomically and trophically highly resolved data set on forest soil invertebrates (Nematoda, Acari, Enchytraeidae, Gastropoda, Myriapoda, Isopoda, Araneae, Insecta) from the Kampinos National Park, Poland (Hoste-Danyłow 2013; Hoste-Danyłow et al. 2013). As data were compiled during six consecutive sampling seasons we were able to study temporal differences in population energy use after phylogenetic correction. Particularly we ask:

1. Does metabolic and biomass—body weight (BWR) scaling of soil invertebrates vary in time, and if so, to what degree?
2. Do these scaling patterns confirm EER predictions?
3. Does phylogenetic correction influence the results?
4. Do soil taxa differ in allometric scaling patterns?

## Materials and methods

### Data

From 2009 to 2011 we studied the soil fauna of a 10-m × 20-m plot located in a 40- to 50-year-old deciduous forest of Kampinos National Park (Poland). The tree and shrub layers covered about 70 % of the plot surface and were dominated by *Quercus robur*, *Betula pendula*, and *Frangula alnus*. *Poa trivialis*, *Agrostis alba*, *Juncus effusus* and *Deschampsia caespitosa* were most abundant in the well-developed herb layer (80–85 % cover).

We quantitatively sampled the soil fauna during six sampling seasons in August and October 2009; April, July, and October 2010; and in May 2011. For each group of organisms core samples were taken close by in ten randomly chosen 1-m^2^ quadrats (below referred to as single samples). To minimize temporal autocorrelation we used different quadrats in each sample session. Each sample was taken to a depth of 10 cm. Nematoda were sampled using a 1.8-cm-diameter corer and were extracted using the Whitehead and Hemming modification of the Baermann method (Whitehead and Hemming 1965). The mesofauna was sampled using a 3.5-cm-diameter corer and extracted using a MacFadyen high-gradient canister extractor (MacFadyen 1961) in the case of springtails and mites and using the O’Connor modification of the Baermann funnel (O’Connor 1955) in the case of enchytraeids. The macrofauna was hand-sorted from 30-cm × 30-cm leaf litter and upper soil samples (Hoste-Danyłow 2013; Hoste-Danyłow et al. 2013). Additionally, we hand-sorted 50-cm × 50-cm surface samples directly in the field to include fast-moving, large invertebrates. All collected individuals were measured and identified by one of us to the species level (cf. Electronic supplement for raw data and information on identification). We estimated body weights either by direct measurements after 48 h at 60 °C or from standard literature regressions (cf. Hoste-Danyłow 2013 and Hoste-Danyłow et al. 2013 for detailed descriptions). In total, our data set contains more than 77,000 individuals from 606 morpho-species (Hoste-Danyłow et al. 2013).

### Statistical analysis

For each sample and each species we estimated population energy use (*M* × *D*) from the product of population density (*D*; individuals m^−2^) and temperature-adjusted individual metabolic rate (*M*; mm^3^ O_2_ × h^−1^). Basal metabolic rates (*M*_0_) were derived from a variety of taxon-specific literature sources compiled by Ehnes et al. (2011), Hoste-Danyłow (2013), and Hoste-Danyłow et al. (2013). They were in all cases adjusted to environmental temperatures according to published taxon-specific *Q*_10_ factors given in Table 1 of Hoste-Danyłow et al. (2013). Population biomass (*B*) was derived from the product of average individual body weight (*W*) and *D*.

**Table 1:** Average species richness and average density ranges (individuals m^−2^) per sample of decomposers, phytophages, and predators in the ten samples in the six sampling seasons

Sampling season	Decomposers		Phytophages		Predators	
	Species richness	Density range	Species richness	Density range	Species richness	Density range
August 2009	55.6	6.07 × 10^7^	75.9	9.13 × 10^7^	128.7	9.14 × 10^6^
October 2009	20.7	1.24 × 10^7^	20.7	4.42 × 10^2^	19.7	3.19 × 10^4^
April 2010	160.8	1.05 × 10^8^	88.1	1.15 × 10^7^	23.9	2.13 × 10^6^
July 2010	27.5	4.41 × 10^5^	33.9	6.44 × 10^6^	2.3	3.13 × 10^4^
October 2010	14.5	1.39 × 10^7^	8.9	1.53 × 10^4^	30.6	7.24 × 10^2^
May 2011	26.3	5.21 × 10^6^	34.9	2.52 × 10^2^	39.9	8.22 × 10^5^

To account for phylogenetic non-independence of data points we used the methods of Ricotta et al. (2012) and Ulrich and Fattorini (2013) and calculated the dominant eigenvector of the taxonomic distance matrix of all species found. Species distances were assessed by the number of nodes separating the species (Ulrich and Fattorini 2013). This vector explained 94 % of the variance in taxonomical distance. Swenson (2009), Ricotta et al. (2012) and Ulrich and Fattorini (2013) showed that taxonomic distances are a sufficient proxy to true dated phylogenetic distances and can be used for phylogenetic inference. We used ordinary least squares regression to estimate, for each feeding guild and sampling season, the slopes of the allometric regressions between average log-transformed individual body weight and population abundances (DWR slopes), biomasses (BWR slopes), and temperature correlated metabolic rates (MWR slopes), in each case using the phylogenetic eigenvector as covariate. Subsequently, we performed general linear modelling (orthogonal sums of squares) as implemented in Statistica 7.0 to relate estimated phylogenetically corrected slope values to species richness, log-transformed body weight range (WR; =maximum weight/minimum weight), sampling season, and feeding guild. To account for possible temporal non-independence of sample plots, sampling season entered the model as a random effect. Errors and error bars in the figures always refer to 2 SEs.

## Results

### General results

**Fig. 1:**
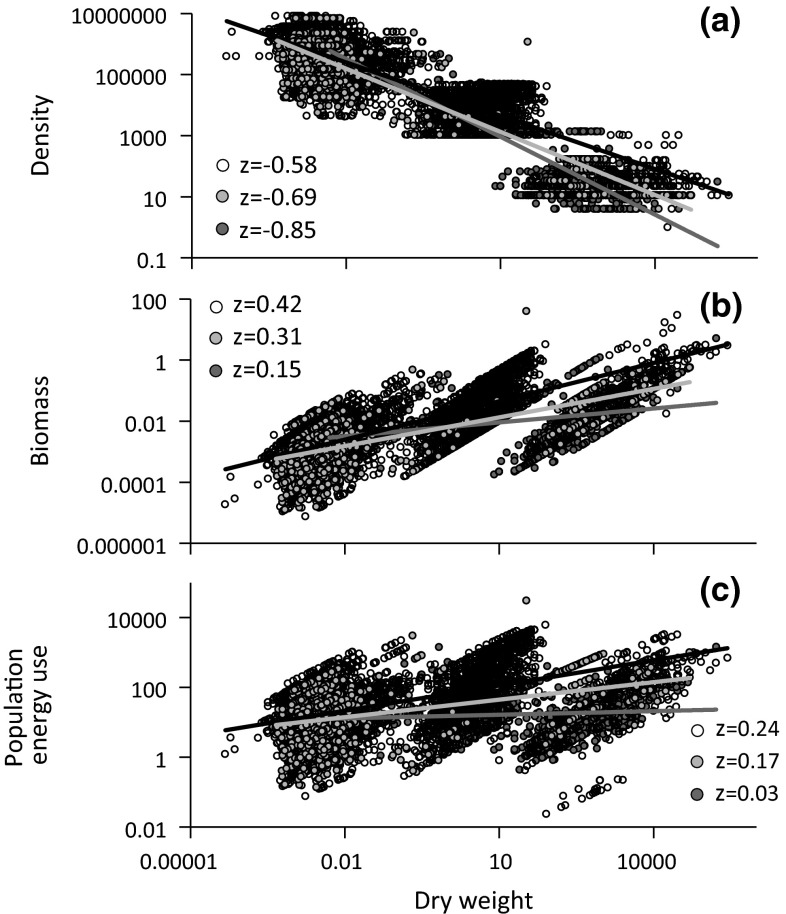
Density (individuals m^−2^) decreases (**a**) and total population biomass (g × m^−2^) (**b**) and temperature-corrected population energy consumption (mm^3^ O_2_ × h^−1^×m^−2^) (**c**) increase with individual dry weight (μg) of predators (*red dots*), phytophages (*green dots*), and decomposers (*open black dots*). Ordinary least squares (OLS) regressions: **a** *r* ^2^ = 0.73 (*red*), *r* ^2^ = 0.80 (*green*), *r* ^2^ = 0.73 (*black*); **b** *r* ^2^ = 0.08 (*red*), *r* ^2^ = 0.50 (*green*), *r* ^2^ = 0.59 (*black*); **c** *r* ^2^ = 0.01 (*red*), *r* ^2^ = 0.18 (*green*), *r* ^2^ = 0.25 (*black*) (color figure online)

Species richness and the range of density considerably varied between sampling seasons and feeding guilds (Table 1) and appeared to be highest in April and August and lowest in July. When calculated over all sampling seasons (Fig. 1a), DWR slopes were lowest for predators (*v* = −0.85 ± 0.02) and highest for decomposers (*v* = −0.58 ± 0.02). Slopes significantly differed between feeding guilds (pair-wise* t*-tests, *P* < 0.001). Population biomass increased in all feeding guilds with body weight (Fig. 1b). This increase was the lowest in predators (BWR slope *y* = 0.15 ± 0.02) and the highest in decomposers (*y* = 0.42 ± 0.02). In turn, population energy consumption was roughly stable in predators (MWR slope *z* = 0.04 ± 0.04) while still increasing with body weight in phytophages (*z* = 0.17 ± 0.04) and decomposers (*z* = 0.24 ± 0.01).

**Fig. 2:**
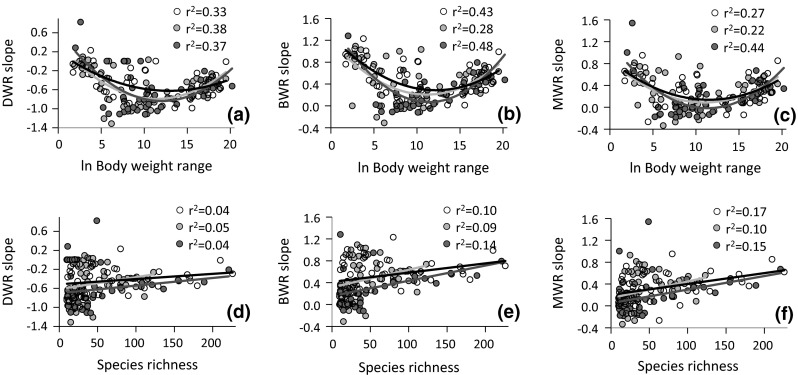
Dependence of density—body weight (*DWR*) (**a**, **d**), biomass—body weight (*BWR*) (**d**, **e**) and metabolic rate—body weight (*MWR*) (**c**, **f**) slopes for each sample (*n* = 157) on the range of body weight (**a**–**c**) and species richness (**d**–**f**) of decomposers (*open circles*), phytophages (*green circles*), and predators (*red circles*). **a**–**c** Quadratic OLS regression terms are significant at *P* < 0.001; **e**, **f** linear regressions are significant at *P* < 0.01 (color figure online)

When calculated for each sample (Fig. 2), DWR, BWR and MWR slopes appeared to be highly variable but were always lower at intermediate body weight ranges (Fig. 2a–c) and increased with species richness (Fig. 2d–f). At the intermediate body weight range from WR = 1 × 10^3^ to WR = 3 × 10^6^, MWR slopes of phytophages (*z* = 0.13 ± 0.14) and predators (*z* = 0.04 ± 0.06) did not significantly differ from zero at *P* < 0.05, while those of decomposers (*z* = 0.23 ± 0.10) did.

### Temporal patterns in energy consumption

**Fig. 3:**
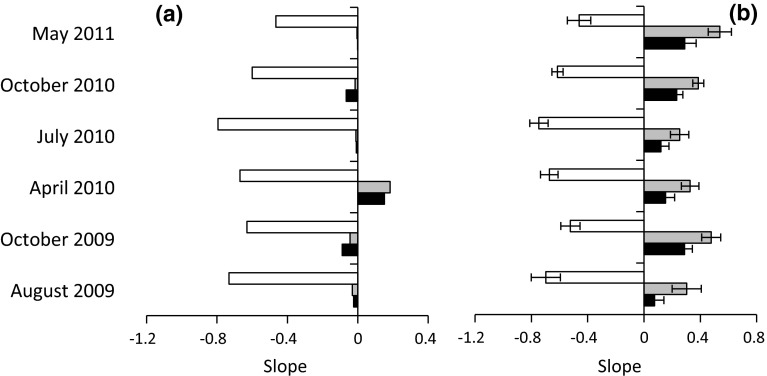
Temporal variability of the slopes of the DWR (*white bars*), BWR (*grey bars*), and MWR (*black bars*) across all feeding guilds and samples (**a**) and average values of the ten samples in each sampling season (**b**) (*error bars* denote 2 SEs). For abbreviations, see Fig. 2

DWR, BWR, and MWR slopes appeared to be variable in time (Fig. 3). This variability was sample-scale dependent. When calculating scaling slopes across trophic groups, slopes were less variable in time when pooling all ten single samples per sampling season (Fig. 3a) than when calculating slopes from sample averages (Fig. 3b). Cross-feeding group pooled samples of BWR (*y* = 0.02 ± 0.06) and MWR (*z* = 0.01 ± 0.06) slopes did not significantly differ from zero (Fig. 3a). When calculated from single samples (Fig. 3b) BWR (*y* = 0.38 ± 0.08) and MWR (*z* = 0.19 ± 0.06) slopes were significantly positive. On average, cross-feeding group DWR slopes were lower, but did not significantly deviate from the predicted −¾ value (*v* = 0.65 ± 0.08) at the 5 % error level. When calculated from single samples in three of six sampling seasons (August 2009, April and July 2010; Fig. 3b) DWR slope *v* did not significantly deviate from the predicted −¾ value.

**Fig. 4:**
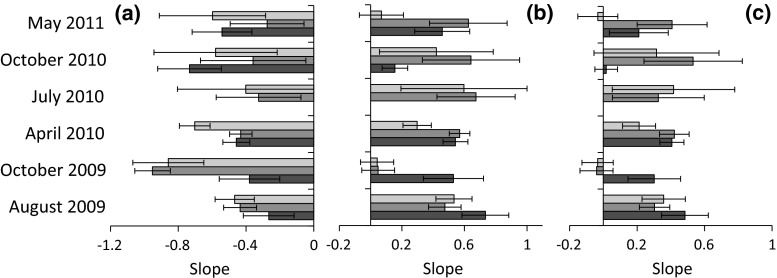
Temporal variability of the slopes of the DWR (**a**), BWR (**b**), and MWR (**c**) of predators (*red*), phytophages (*green*), and decomposers (*grey*). *Error bars* denote 2 SEs. For abbreviations, see Fig. 2 (color figure online)

In contrast to the cross-feeding group data the feeding types constantly differed from Kleiber’s rule (Fig. 4a) and EER (Fig. 4b, c) expectations. One-way ANOVA detected for all three feeding guilds significant (*P* < 0.001) differences in slope values between sampling seasons. These differences remained after accounting for differences in species richness and body weight range (Table 2). Weight range, species richness and sampling season explained 55 % of the variance in the MWR, and 64 % in the BWR slope. After accounting for species richness, sampling season and weight range, the feeding guild had only a minor influence on slope values (Table 2).

**Table 2:** General linear modelling (orthogonal sums of squares) detected significant differences in biomass—body weight (*BWR*), and metabolic rate—body weight (*MWR*) slopes between sampling seasons (random effect) even after correcting for the influence of feeding guild (fixed effect), species richness and ln-transformed body weight ranges (metric variables)

Factor	*df*	BWR	MWR
Ln weight range	1	21.6***	17.4***
Squared ln weight range	1	10.4**	8.7**
Species richness	1	7.8**	5.1*
Sampling season	5	7.7**	5.1**
Feeding guild	2	3.5	0.9
Sampling season × Feeding guild	9	1.1	1.1
Error	137		
*r* ^2^ (whole model)		0.64***	0.55***

Parametric *F*-values

* *P* < 0.05, ** *P* < 0.01, *** *P* < 0.001

### Species groups in abundance–weight space

As a side effect, our study identified three groups of soil species separated in body weight and species abundance space (Fig. 1a). These groups coincide roughly with the division into micro- (from 10^−5^ to 10^−1^ μg), meso- (from 10^−1^ to 10^2^ μg), and macrofauna (10^2^–10^5^ μg). Within each group and irrespective of trophic guild, did not correlate with body weight (all six *P* > 0.05). Consequently, according to Eq. 3. population biomass (Fig. 1b) and energy use (Fig. 1c) increased with body weight approximately to the average rate of the underlying individual metabolic rate—body weight relationships (Eq. 1): slope *u* = 0.82 ± 0.01 (decomposers), *u* = 0.85 ± 0.01 (phytophages), and *u* = 0.89 ± 0.01 (predators).

## Discussion

### Temporal variability in energy equivalence

As most work on energy equivalence used temporal point data to assess population energy use across a range of body weights (cf. Savage et al. 2004; Meehan 2006a, b; Hechinger et al. 2011; Ehnes et al. 2014), we tried to assess the temporal variability in the distribution of energy use. Our results on the temporal variability of allometric scaling of density, biomass, and energy use (Table 2) corroborate previous findings that empirical patterns obtained from small-scale spatial or temporal observations deviate from simplified theoretical models that assume a prevalence of ¾ scaling laws (Brown et al. 2004; Savage et al. 2004) and energy equivalence (Damuth 1987; Allen et al. 2002).

Energy use is generally measured indirectly by multiplying individual metabolic rates and population density (e.g. Allen et al. 2002; Brown et al. 2004; Ehnes et al. 2014). As metabolic rates nearly always stem from literature data and are assumed to be only dependent on body temperature (Brown et al. 2004), temporal variability of estimated population energy use should consequently be mainly caused by the temporal variability in density. In nearly all soil populations, density is highly variable (e.g. Wolters 1998). If populations fluctuate independently of each other statistical averaging should cause derived regressions with body weight to return repeatable slopes (cf. Blackburn and Gaston 1997). However, density fluctuations are rarely independent of each other but are temporally correlated (Liebhold et al. 2004) triggered mainly by climatic conditions (Andrewartha and Birch 1954; Menge and Sutherland 1987) and trophic cycling (Chase 1996; Ovadia and Schmitz 2004). Thus, at any given point in time we expect allometric regressions that include density terms to deviate from predicted average values. At best, we expect the regression parameters, particularly the slope, to have a central tendency to converge towards the expected values (Farrell-Gray and Gotelli 2005; Hayward et al. 2009). This was indeed the case. At each single point in time nearly all BWR and MWR regression slopes were significantly positive (Figs. 3b, 4) while they became increasingly scattered around the predicted zero value after temporal averaging (Fig. 3a). Our results thus corroborate findings from the spatial scaling of energy equivalence (e.g. Griffiths 1992; Arneberg et al. 1998; Cyr 2000; Meehan et al. 2006) and show that this scale dependence also regards the temporal aspect. We argue that temporal variability in population energy use is the natural consequence of the temporal variability in abundance that is the major input variable of all population energetic calculations.

Another important factor in allometric regression is the variance of the x-axis variable, in the present case the body weight. This does not regard measurement uncertainties that would demand the use of major axis or reduced major axis regressions (Smith 2009) but the total range in density. Arneberg et al. (1998), Griffiths (1992), and Hayward et al. (2009) have demonstrated already that decreasing body weight ranges make slope estimates more variable and argued that different body weight ranges might explain at least part of the contrary results obtained from different studies on energetic scaling. Our results partly confirm these arguments. Slope estimates obtained from single samples, sampling seasons, and guilds differed systematically from those obtained by pooled data. Pooled data were always closer to the theoretical predication of energy equivalence (Figs. 1, 3) while single-sample data returned an increase of population energy use with body weight (Figs. 3, 4). However, we did not find a simple weight range—EER consistent relationship. To our surprise, deviations from theoretical expectation were lowest at the intermediate body weight range (Fig. 2). According to our argument above this range is equivalent to the steepest slope of the DWR. Given that shallow DWR slopes are expected for small weight ranges, the shallow DWR slopes at large body weight ranges need explanation. Apparently, large-bodied species have higher average abundances than predicted from a simple allometric scaling law. This points to nonlinear DWR scaling as had previously been observed in mammals by Silva and Downing (1995) and for various taxa by Blackburn and Gaston (1997), but still awaits a functional explanation [but see Mulder and Elser (2009) for a possible role of phosphorus input on DWR slopes of soil animals].

Being closer to expectation does not necessarily mean being in agreement with expectation. Our regression analyses that accounted for differences in species richness and body weight range returned a significant temporal variability in BWR and MWR regression slopes (Table 2; Fig. 4). As our data were taken in consecutive seasons, seasonal variability in species composition, weather regimes, and soil properties are most likely to influence patterns in population abundance and energy use. Possibly, abiotic factors that induced changes in trophic structure are a major force in the observed variability in energy use. Soil food webs are known to be sensitive to changing pH, temperature and moisture regimes (Berg and Bengtsson 2007). Such variability necessarily translates into differential energy transfer through the web. If this argument is correct, we might speculate that population energy use is regulated in a density-dependent manner similar to population densities. In this case we might be able to identify the steady-state pattern of the MWR from population abundance modelling within a Lotka-Volterra framework. Indeed, a recent analysis of Henderson and Magurran (2014) on the variability of fish population biomasses indicates that biomass (as a proxy of energy use in poikilothermous species) might indeed be density dependent. However, six sampling seasons are too short a time scale for any correlation of patterns with abiotic conditions. Therefore, our results clearly indicate the need for long-term data on population abundances to assess patterns in the variability of metabolic scaling.

### Trophic position and energy equivalence in soil animals

We found trophic position to have a strong influence on the relationship between total population energy flux and body mass (Figs. 1, 4). Deviation from energy equivalence increased with trophic rank being highest in decomposers and lowest in predators. Similar results have been obtained by Marquet et al. (1995), Russo et al. (2003), and Ehnes et al. (2014). As also found by Ehnes et al. (2014), phytophages were intermediate between decomposers and predators (Fig. 1). These results contrast to simple trophic-level models of energy use (Hechinger et al. 2011) and resource thinning (Brown and Gillooly 2003, Long et al. 2006) that predict decreasing DWR slopes with increasing trophic position. Possibly, these models but also a simplified trophic classification might underestimate the variability in trophic relationships, particularly the intraspecific variability and the effect of omnivores on energy transfer through food chains (Reuman et al. 2009).

### Species clusters in density–weight space

Our study identified three distinct species clusters in the weight—abundance space (Fig. 1a) in each case comprising decomposers, phytophages, and predators. Figure 1f of Ehnes et al. (2014) indicates a similar, although weaker, clustering in German temperate forests. The three groups roughly coincide with the distinction into micro-, meso-, and macrofauna. As we included nearly all major invertebrate orders in the present study and body weight widely overlapped among the clusters this result is apparently not caused by a taxonomic bias. It also does not stem from a bias in the density estimates across species body weights as our data are based on a mix of well-introduced quantitative extraction methods that overlapped across size groups.

The most parsimonious explanation for the observed pattern is that each of the three groups contains independent clusters of food webs with basal species having different body weights. Body weight generally, but by no means always, increases, and abundance and transferred energy decrease along trophic chains (Lawton 1999; Brose et al. 2006). In the soil system a variety of complex food webs are based on different basal phytophagous or decomposer species belonging to the micro-, meso-, and macrofauna (Schaefer 1990; Wardle 2002). In this case metabolic and abundance scaling might strongly vary among webs causing statistical averaging to override the scaling patterns. This might result in the observed independence of abundance and body weight within each cluster of species. Clearly the observed clusters need further study with respect to food web structure and energy transfer.

### The influence of phylogeny on allometric scaling

Our study used taxonomic relatedness and eigenvector mapping to account for phylogenetic non-independence. Although this was not the major focus of our study, we note that slope values calculated with and without correction were only moderately correlated (on average *r* = 0.63) and taxonomic relatedness explained in 38.7 % of regressions a significant part of variance at the 5 % error level and in even 90.0 % at the relaxed 10 % error level. Similar results have been reported by Duncan et al. (2007), Clarke and Rothery (2008), and Sieg et al. (2009), and indicate that phylogenetic effects might have a large impact on inferences of metabolic scaling exponents (Capellini et al. 2010). These and our results do not corroborate earlier claims that phylogenetic non-independence biases only statistical significance but not the estimates of regression parameters (reviewed in Glazier 2005).

However, this does not mean that phylogenetic correction necessarily reconciles data with EER predictions, as found by George-Nascimento et al. (2004) and Raichlen et al. (2011). With respect to the present soil fauna only 48 % of the phylogenetically explicit slope values of MWR were more in accordance with ERR than the uncorrected values, while 29 % were less in accordance. In 23 % of samples neither slope differed (*∆*_slope_ < 0.01). Therefore, meta-analytical and empirical studies rejecting (e.g. Dodds et al. 2001; White et al. 2007; Makarieva et al. 2008) as well as studies accepting energy equivalence (e.g. Brown et al. 2004, Savage et al. 2004) might have come to biased conclusions while not accounting for phylogenetic independence (Duncan et al. 2007).

Our results also suggest that taxonomic grouping might not be an appropriate way to account for phylogenetic non-independence (cf. Ehnes et al. 2011, 2014). Taxonomic grouping inevitably reduces species richness and body weight range, the two variables that influenced most the density-weight relationship (Table 2). As a consequence DWR slopes tend to be shallower causing an increase in the derived MWR slopes, as observed for vertebrates by Brown and Maurer (1988), Damuth (1991), and Munn et al. (2013), for eusocial insects by DeLong (2011), and for soil invertebrates by Ehnes et al. (2014). We conclude that the slopes observed in these papers might have been more in accordance with EER when using explicit phylogenetic regression methods.

### Conclusion

Our work adds the temporal aspect to the growing number of studies reporting taxon, feeding guild, and spatial variation in the metabolic scaling of animals. Although we demonstrate a high temporal variability in population energy use and a general increase in energy use with species body weight, our results do not exclude the possibility of energy equivalence as a central tendency in time. Our results highlight the need for long-term field studies on population densities and energy use to derive unequivocal conclusions about metabolic scaling within ecosystems.

#### Author contribution statement

A. H.-D. developed the field study design, collected the soil data, and provided the data base. A. H.-D., K. F.-K., I. H., K. I.-M., I. O., and J. W. identified the material. A. H.-D. and W. U. performed the phylogenetic and regression analyses. W. U. wrote the first draft of the manuscript, and A. H.-D. and W. U. contributed to revisions.

## Electronic supplementary material

Supplementary material 1 (XLS 4061 kb)
